# Differences Between Interictal and Ictal Generalized Spike-Wave Discharges in Childhood Absence Epilepsy: A MEG Study

**DOI:** 10.3389/fneur.2019.01359

**Published:** 2020-01-24

**Authors:** Qi Shi, Tingting Zhang, Ailiang Miao, Jintao Sun, Yulei Sun, Qiqi Chen, Zheng Hu, Jing Xiang, Xiaoshan Wang

**Affiliations:** ^1^Department of Neurology, The Affiliated Brain Hospital of Nanjing Medical University, Nanjing Medical University, Nanjing, China; ^2^MEG Center, The Affiliated Brain Hospital of Nanjing Medical University, Nanjing, China; ^3^Department of Neurology, Nanjing Children's Hospital, Nanjing, China; ^4^Division of Neurology, MEG Center, Cincinnati Children's Hospital Medical Center, Cincinnati, OH, United States

**Keywords:** childhood absence epilepsy (CAE), generalized spike-wave discharges (GSWDs), interictal and ictal period, Magnetoencephalography (MEG), multi-frequency bands

## Abstract

**Purpose:** To investigate the differences between interictal and ictal generalized spike-wave discharges (GSWDs) for insights on how epileptic activity propagates and the physiopathological mechanisms underlying childhood absence epilepsy (CAE).

**Methods:** Twenty-five patients with CAE were studied using magnetoencephalography (MEG). MEG data were digitized at 6,000 Hz during the interictal and ictal GSWDs. GSWDs were analyzed at both neural magnetic source levels and functional connectivity (FC) in multifrequency bands: delta (1–4 Hz), theta (4–8 Hz), alpha (8–12 Hz), beta (12–30 Hz), gamma (30–80 Hz), ripple (80–250 Hz), and fast ripple (250–500 Hz). Brain FC was studied with the posterior cingulate cortex/precuneus (PCC/pC) as the seed region.

**Results:** The magnetic source of interictal GSWDs mainly locates in the PCC/pC region at 4–8 and 8–12 Hz, while that of ictal GSWDs mainly locates in the medial frontal cortex (MFC) at 80–250 Hz. There were statistically significant differences between interictal and ictal GSWDs (*p* < 0.05). The FC network involving the PCC/pC showed strong connections in the anterior-posterior pathways (mainly with the frontal cortex) at 80–250 Hz during ictal GSWDs, while the interictal GSWDs FC were mostly limited to the posterior cortex region. There was no significant difference in the magnetic source strength among interictal and ictal GSWDs at all bandwidths.

**Conclusions:** There are significant disparities in the source localization and FC between interictal and ictal GSWDs. Low-frequency activation in the PCC/pC during inhibition of seizures possibly relates to the maintenance of consciousness during interictal GSWDs. High-frequency oscillations (HFOs) of the MFC during CAE may associate with the inducing or occurrence of GSWDs. Weakened network connections may be in favor of preventing overexcitability and relates to the termination of GSWDs.

## Introduction

Childhood absence epilepsy (CAE) is a type of idiopathic generalized epilepsy (IGE) characterized as episodes of unresponsiveness and generalized spike and wave discharges (GSWDs) on scalp electroencephalogram (EEG) of 3–4 Hz (1). When the duration of GSWDs was more than 4 s, the typical symptoms can be observed clinically, whereas the GSWDs lasting <4 s (interictal GSWDs) are usually asymptomatic (2). The seizures are thought to be relative to abnormal interplays in thalamocortical network (3–5), which affects patient awareness (6). The global workspace (GW) theory proposes that information is processed in an unconscious manner when numerous modular cerebral networks are synchronized activity. However, consciousness would be formed if the corresponding neural population is mobilized through top-down attention amplification into a state of self-sustaining activity, which involves many neurons distributed throughout the brain (7). The consciousness system has cortical and subcortical components, most notably being the medial, lateral, and orbital frontal cortex; anterior and posterior cingulate cortex (ACC, PCC); medial parietal (precuneus, pC) cortex; lateral temporal-parietal association cortex; basal forebrain; thalamus; and the upper brain stem activating systems (involved in higher order association) (8). Network inhibition hypothesis proposes that the subcortical arousal systems are inhibited by spread of seizure-related activity, which generates sleep-like or coma-like slow-wave activity in the bilateral frontoparietal association cortex and leads to disturbance of consciousness (1). A subset of these localized in the medial frontal cortex (MFC), PCC/pC, and inferior temporal makes up the default mode network (DMN), a state of resting brain function (9). These brain areas usually show decreased activity during attention-demanding tasks and become increasingly active during inattention to mental tasks or the external environment (10, 11). Also, the prefrontal cortex, anterior cingulate, and parietal cortex are important in creating the assumptive brain-scale workspace. Currently, the most accepted generation mechanisms of SWDs are cortico-reticular theory and cortical focus theory. The former assumes that the SWDs are closely related to the thalamocortical mechanism that generates sleep spindles (5, 12). The latter assumes that there is a focal area (pC, prefrontal and parietal cortex regions) active before occurrence of SWDs (13). Then cortical and thalamic alternating resonance (the cortical and thalamus in turn drives each other) produces SWDs. The integrity of the network is a prerequisite in both theories. Accordingly, the DMN, which integrates cognitive and emotional processing (14), is disturbed during absence seizures (15). Previous works studying GSWDs with EEG-fMRI have shown that the frontal, parietal regions, thalamus, and DMN (9) are changed with blood oxygen level-dependent (BOLD) signals (9, 16–18) in absence seizures (19–23). Disorders of consciousness may thus be caused by selective bilateral cortical and subcortical networks (5, 24) through abnormal patterns of neural activity (1).

It is well-known that typical GSWDs originate from paroxysmal oscillations in corticothalamic networks, but the underlying mechanism is still unclear (5, 25, 26). The appearance of BOLD changes related to GSWDs in the PCC/pC may imply that this region has an important role in the pathogenesis of CAE (27, 28). This might indicate that alteration of the PCC/pC with levels of activity is relevant to the changes of GSWD. However, the exact interplay between the cortical and subcortical structures should be further explored. The present study aimed to investigate the difference of magnetic source localization and FC between the interictal and ictal GSWDs in various frequency bands in CAE patients using MEG, which has better spatial resolution comparing with EEG and higher temporal resolution than magnetic resonance imaging (MRI) (29).

To the best of our knowledge, there have been some studies on the frequency-dependent nature of CAE. The connectivity networks and sources during the evolvement of SWDs with spatial and temporal profiles were described (30). Tenney et al. revealed that different ictal connectivities in pretreatment were related with response to antiepileptic treatment (31). And the changes of the effective connectivity (EC) network in specific frequency during interictal period were reported by Wu et al. (32). However, in our study, we try to investigate the differences between interictal and ictal GSWDs using FC and sources. The findings may contribute to our understanding of the physiopathological mechanism underlying consciousness disorders and mechanisms that sustain epileptic discharge.

## Methods

### Subjects

Forty-two CAE patients aged 5–14 years old were recruited from the Department of Neurology at the Nanjing Children's Hospital and Nanjing Brain Hospital during March 2012–December 2018. The research received approval from the medical ethics committees of Nanjing Medical University, Nanjing Brain Hospital and Nanjing Children's Hospital. The inclusion criteria were (1) typical CAE consistent with the International League Against Epilepsy Seizure Classification (2017) diagnosed by a neurologist; (2) bilaterally synchronous 3–4 Hz SWDs on a normal background EEG; (3) normal physical and neurological examination; (4) no abnormal brain MRI; and (5) head movement <5 mm during MEG recordings. Patients were excluded if they met any of the following criteria: (1) history of other major neurologic or psychiatric diseases or severe systemic disease; (2) having metal implants that interfere with MEG data, such as pacemakers or cochlear devices; (3) incompatibility in keeping head motionless during MRI scans or MEG recordings. Of the 42 CAE patients recruited, 17 were excluded due to excessive head movements or absence of GSWDs. Accordingly, 25 subjects (7 males, 18 females) fulfilled our inclusion criteria and were eligible for the study. This study was approved by the medical ethics committees of the Nanjing Medical University, Nanjing Children's Hospital, and Nanjing Brain Hospital. All subjects and their guardians were informed about the purpose of this study and signed their written informed consent.

### MEG

All participants were asked to reduce sleeping time before MEG recordings to raise the probability of absence of seizures during data recordings. MEG data were recorded in a magnetically shielded room with a whole-head CTF MEG system with 275 channels (VSM Medical Technology Company, Canada) at the MEG Center at the Nanjing Brain Hospital. Background noises were routinely measured by MEG before the experiment in an empty room. Before data acquisition, three coils were attached to the pre-auricular points and nasion of each subject to aid head localization relative to the MEG coordinate system. All subjects were asked to stay still (avoiding swallowing or teeth clenching) and close their eyes slightly in a supine position. Participants were monitored with an audiovisual system during MEG recording. Tolerance for head movement before and after acquisition was limited to 5 mm for each recording. Head localization was accurate to within 1 mm. The sampling rate was 6,000 Hz with noise cancelation of third-order gradients. A minimum of six consecutive epochs, each with a duration of 2 min, were recorded for each subject. If no GSWDs were recorded in the first three files, we will make the patient blow a slip of paper for a while to hyperventilate, which will increase the chance of seizure.

### MRI

All 25 subjects have informed consent to undergo MRI with a 3.0T scanner (Siemens, Germany). Three fiduciary markers were attached to the same pre-auricular points and nasion used for MEG. All anatomical landmarks digitized during MEG were identifiable in the MRI.

### Data Analyses

**Figure 1:**
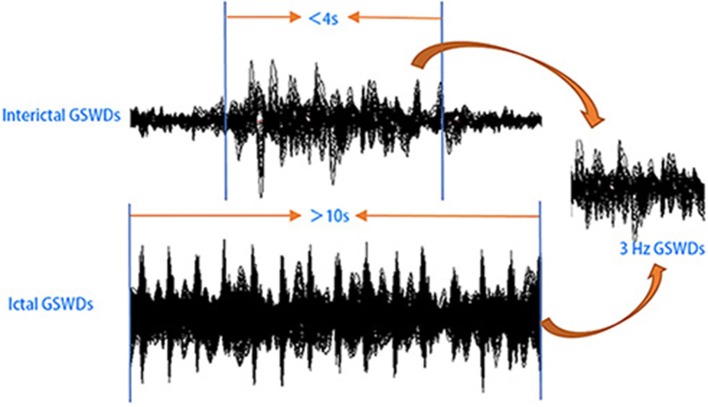
Schematic of magnetoencephalography (MEG) data analysis. MEG waveforms were recorded from 25 patients with childhood absence epilepsy (CAE). MEG data segments with generalized spike-wave discharges (GSWDs) <4 s or >10 s were selected for further analysis.

Informed by previous studies (32–35), MEG data without noise or artifacts (>6 pT) were filtered with a band pass filter of 1–4 Hz. And the MEG waveform segments showing GSWDs indicative of absence epilepsy were thereafter marked. In this study, we try to investigate the differences between interictal and ictal GSWDs in CAE. Only the GSWDs with an interictal period of <4 s (2, 21) and an ictal period of more than 10 s were selected for analysis. For segments longer than 10 s, the audiovisual system recordings were checked for subject unconsciousness to verify ictal periods. Recording the start and end times of seizure, then choosing 3 s in the middle of the seizure, which must be in the state of unconsciousness and applying it to all frequency bands for further study (because sometimes when patients presented with clinical manifestations of absence epilepsy, 3–4 Hz GSWDs would appear on the MEG earlier or later than it, so we choose the middle data of the ictal period, which would ensure that the selected study data were in the ictal period of absence epilepsy). Interictal segments were selected on both clinical observation of consciousness and presence of 3 Hz GSWDs in MEG as per the following three-step protocol: (1) waveform segments with GSWDs of 3 Hz shorter than 4 s were identified; (2) consciousness was observed via audiovisual recordings; (3) checking for the presence of ictal GSWDs before or after the chosen segments. If there was at least 30 s away from ictal segments, then the waveform was selected as interictal GSWDs data for the following analysis. An overview of MEG acquisition is presented in Figure 1.

MEG signals were filtered into bandwidths of 1–4 Hz (delta), 4–8 Hz (theta), 8–12 Hz (alpha), 12–30 Hz (beta), 30–80 Hz (gamma), 80–250 Hz (ripple), and 250–500 Hz (fast ripple). A notch filter of 50 Hz and harmonics were applied to eliminate power-line noise. Bandwidth segments were chosen based on our own and others' previous studies of epilepsy (35, 36). We calculated the accumulated source localization (ASI) and FC during the interictal and ictal GSWDs for 3 s time window in seven frequency bands from all patients.

#### ASI

ASI was defined as the volumetric summation of source activity over a period of time. ASI was localized to correlated sources using node-beam lead fields (37). Given that each node-beam lead field represents a form of either source-beamformer or subspace solution, the ASI had multiple source beamformers to separate correlated sources. The mathematical algorithms and validations have been described in detail (37, 38). Measurements of brain activity were enhanced by inclusion of the source activity strength. Source location was quantitated in a three-dimensional (3-D) coordinate system where the X, Y, and Z axes represented each of the three MEG fiducial points. A MEG Processor was used in the measurements of neuromagnetic source strength (37).

#### FC

FC was analyzed at the source level (35, 37). Each source was computed by virtual sensor waveforms using ASI algorithms. The source neural networks were estimated from the signal correlation of each pair of virtual sensors in the interictal and ictal GSWDs. Specifically, by calculating correlation coefficients, the relationship between virtual sensor signals from two source pairs is defined as follows:

(1) $$\begin{array}{l} \text{R}\left({\text{X}}_{\text{a}},{\text{X}}_{\text{b}}\right)=\frac{\text{C}\left({\text{X}}_{\text{a}},{\text{X}}_{\text{b}}\right)}{\text{S}{\text{X}}_{\text{a}},{\text{X}}_{\text{b}}} \end{array}$$

where R (Xa, Xb) represents the correlation of a source pair in two locations (“a” and “b”). The Xa and Xb represent the signals in two sources, which were paired for connection calculation. C (Xa, Xb) represents the mean of two source signals. Sxa and Sxb represent the standard deviation of two source signals. To avoid possible bias, we used the source-level analysis to calculate all possible connections for each two-source pair. Each pair of possible FC distributions of all voxel-based virtual sensors is registered in each participant's MRI (26, 29). To analyze the source connections, MSI-based neural networks were visualized in both axial, coronal, and sagittal views, respectively (39). Red and blue represent the excitatory and inhibitory connections, respectively. A threshold equivalent to *p* < 0.05 was used as a checkpoint to ensure the quality of the data.

(2) $$\begin{array}{l} \text{Tp}=\text{R}\sqrt{\frac{K-2}{1-R^{2}}} \end{array}$$

In Equation (2), Tp is the t value of a correlation, R is the correlation of a source pair, and K is the number of data points for the connection. We used the T_p_ value of *p* < 0.05 as the threshold to obtain the FC network. The PCC/pC was the region of interest (ROI). The ROI was visually defined, and the template was verified by coordinates.

### Statistical Analyses

The chi-squared test (or Fisher's exact test) was used to compare the source localization and FC at low-to-high bandwidth. The Student's *t*-test and Mann-Whitney test were used to compare source strength between interictal and ictal GSWDs for independent samples. A *p* < 0.05 was considered statistically significant. Bonferroni's correction was applied to all *p*-values derived from multiplicity analysis. All statistical analyses were performed with SPSS 25.0 for Windows (SPSS Inc., Chicago, IL, USA).

## Results

The average age of the 25 subjects at CAE onset was 7.7 ± 2.08 years. The average seizure frequency was 7.4 ± 5.36 times per day. Of 57 GSWDs recorded by MEG, 17 incomplete GSWD segments were excluded. Thus, 40 MEG segments, including 20 interictal GSWDs (without conscious disorder) and 20 ictal GSWDs (with conscious disorder), were selected from 25 subjects, with at least one segment and at most two segments of MEG data from each subject. Clinical data are presented in Table 1.

**Table 1:** Clinical data of 25 enrolled subjects with CAE.

**Subject**	**Sex/age** ** (F or M/years)**	**Duration of epilepsy (months)**	**Seizure frequency (times/day)**	**AED at the time of recording**
1	F/7	1	20	None
2	F/6	12	10	None
3	M/7	25	10	VPA and LTG
4	F/10	24	5–7	LTG
5	F/8	3	20	None
6	F/9	60	2–5	None
7	M/8	2	5–8	VPA
8	F/7	4	10	VPA
9	F/5	2	7–8	None
10	F/10	10	4–5	None
11	F/9	3	15–20	None
12	M/14	6	2–3	OXC
13	F/10	11	4–5	None
14	F/5	5	1–2	None
15	F/6.5	4	5–6	None
16	F/5.5	24	6–8	OXC and LTG
17	F/5	2	10	None
18	F/6	12	8–10	VPA
19	F/8	1	4–5	VPA
20	M/7	1	0–1	VPA
21	M/10	72	4–5	VPA
22	F/8	24	0–1	VPA
23	F/6	2	5–10	None
24	M/7.5	5	2–3	VPA
25	M/8	4	10	None

*F, female; M, male; VPA, valproate; OXG, oxcarbazepine; LTG, lamotrigine; CAE, childhood absence epilepsy; AED, antiepileptic drug*.

### Source Localization

#### Delta (1–4 Hz)

The source of interictal GSWDs was mainly localized in the MFC (*n* = 13), PCC/pC (*n* = 9), and parietal-occipital-temporal junction (POT, *n* = 7). Ictal GSWDs were localized in the MFC (*n* = 12), POT (*n* = 5), and middle occipital cortex (MOT, *n* = 5). There were no statistically significant differences between the two groups.

#### Theta (4–8 Hz)

**Figure 2:**
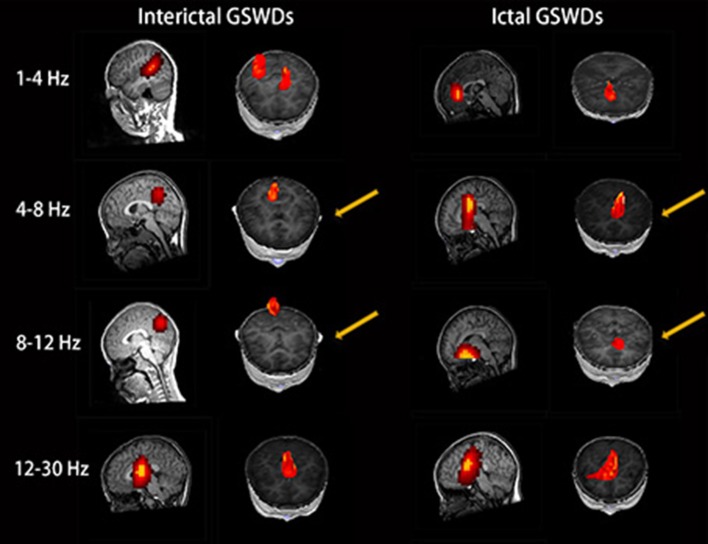
Magnetic source images show the locations of generalized spike-wave discharges (GSWDs) in patients at 1–4, 4–8, 8–12, and 12–30 Hz. Interpatient variation withstanding, the precuneus (pC) is always activated during interictal GSWD at 4–8 and 8–12 Hz. At the same bandwidths, ictal GSWDs are mostly localized in the medial frontal cortex (MFC). Yellow arrows show the differences between the two groups.

The source of interictal GSWDs was mainly localized in the PCC/pC (*n* = 12), MFC (*n* = 9), and POT (*n* = 9). Ictal GSWDs were localized in the MFC (*n* = 14), thalamus (*n* = 6), MOC (*n* = 4), and POT (*n* = 4). There were statistical differences in the PCC/pC localization of interictal and ictal GSWDs (*p* < 0.05) (Figure 2).

#### Alpha (8–12 Hz)

The source of interictal GSWDs was mainly localized in the MFC (*n* = 11), thalamus (*n* = 9), PCC/pC (*n* = 8), and POT (*n* = 8). Ictal GSWDs were mainly localized in the MFC (*n* = 12), POT (*n* = 7), and temporal cortex (*n* = 4). There were statistical differences in the PCC/pC localization of interictal and ictal GSWDs (*p* < 0.005) (Figure 2).

#### Beta (12–30 Hz)

The source of interictal GSWDs was localized in the MFC (*n* = 10), thalamus (*n* = 10), and POT (*n* = 8), whereas ictal group mainly localized in the MFC (*n* = 13), POT (*n* = 7), thalamus (*n* = 7), and temporal cortex (*n* = 7). There were no statistically significant differences between the two groups.

#### Gamma (30–80 Hz)

Interictal GSWDs were mainly localized in the MFC (*n* = 12), thalamus (*n* = 6) and PCC/pC (*n* = 3). Ictal GSWDs were mainly localized in the MFC (*n* = 15), temporal cortex (*n* = 3), thalamus (*n* = 2), and POT (*n* = 2). There were no statistically significant differences in the localization of interictal and ictal GSWDs.

#### Ripple (80–250 Hz)

**Figure 3:**
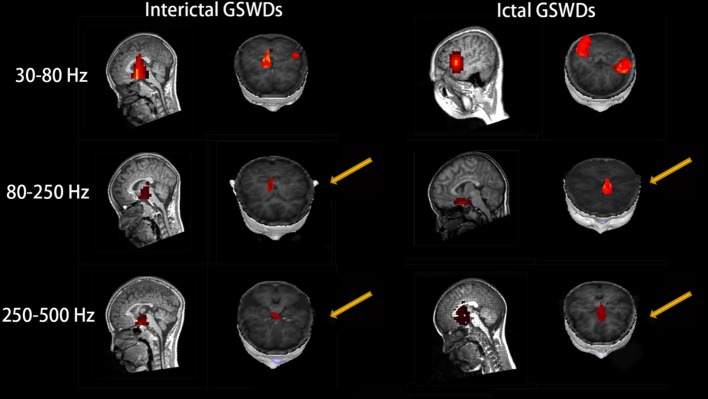
Magnetic source images show the locations of generalized spike-wave discharges (GSWDs) in patients at 30–80, 80–250, and 250–500 Hz. At the 80–250 Hz, medial frontal cortex (MFC) are always activated during ictal GSWDs, while during interictal GSWDs, the locations are almost in the deep brain area (DBA). There are more DBA locations during interictal GSWDs when comparing with ictal GSWDs at 250–500 Hz. The yellow arrows show the differences between the two groups.

Interictal GSWDs were mainly localized in the deep brain area (DBA) (*n* = 8), the MFC (*n* = 4), and temporal cortex (*n* = 4). Ictal GSWDs were localized in the MFC (*n* = 11), DBA (*n* = 5), and temporal cortex (*n* = 5). There were statistical differences in the MFC localization of interictal and ictal GSWDs (*p* < 0.05) (Figure 3).

#### Fast Ripple (250–500 Hz)

Interictal GSWDs were mainly localized in the DBA (*n* = 11), the MFC (*n* = 7), and temporal cortex (*n* = 2). Ictal GSWDs were mainly localized in the MFC (*n* = 12), temporal cortex (*n* = 6), and DBA (*n* = 2). There were statistically significant differences between interictal and ictal GSWDs (*p* < 0.05) (Figure 3).

**Figure 4:**
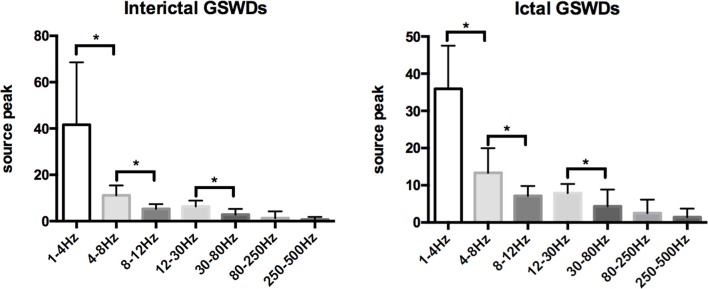
Comparison of neuromagnetic peak source strength for seven frequency bands in interictal and ictal generalized spike-wave discharges (GSWDs). *Statistically significant at *p* < 0.005.

Tables 2, 3 show that the neural magnetic sources were localized to PCC/pC during interictal GSWDs at 4–8 Hz (*p* < 0.05) and 8–12 Hz (*p* < 0.005). In comparison, ictal GSWDs localized in the MFC were mainly observed at 80–250 Hz (*p* < 0.05). There was no statistical difference between the two groups at all other frequency bands (1–4, 12–30, 30–80, 250–500 Hz). Moreover, the strength of brain activity shows that there is no significant difference between the two groups. The measurements of neuromagnetic peak source strength are presented in Table 3. The strength of activity in both groups decreased non-linearly as the frequency bands increased. The strength of activity decreased between 1–4 and 4–8 Hz, 4–8 and 8–12 Hz, 12–30 and 30–80 Hz, but there was no degressive trend between 8–12 and 12–30 Hz, 30–80 and 80–250 Hz, 80–250 and 250–500 Hz in both groups (Figure 4).

**Table 2:** Magnetic source localization during interictal and ictal GSWD.

**Frequency band (Hz)**	**1–4**		**4–8**		**8–12**		**12–30**		**30–80**		**80–250**		**250–500**	
**Group**	**A**	**B**	**A**	**B**	**A**	**B**	**A**	**B**	**A**	**B**	**A**	**B**	**A**	**B**
MFC	13	12	9	14	11	12	10	13	12	15	4^*^	11^*^	7	12
TC	5	3	7	3	3	4	6	7	2	3	4	5	2	6
PCC/pC	9	4	12^*^	3^*^	8^*^	0^*^	3	1	3	0	0	0	0	0
MOC	3	5	1	4	1	1	2	4	1	1	0	0	0	0
TH	6	0	8	6	9	0	10	7	6	2	0	0	0	0
TPJ	2	2	1	1	3	0	2	0	0	0	0	0	0	0
POT	7	5	9	4	8	7	8	7	0	2	0	0	0	0
CE	1	2	2	3	4	1	0	2	1	0	3	2	0	0
DBA	0	0	0	0	0	1	0	0	2	1	8	5	11^*^	2^*^

*GSWD, generalized spike-wave discharge; A, interictal GSWDs; B, ictal GSWDs; MFC, medial frontal cortex; TC, temporal cortex; PCC, posterior cingulate cortex; pC, precuneus cortex; MOC, medial occipital cortex; TH, thalamus; TPJ, temporal-parietal junction; POT, parieto-occipito-temporal junction; CE, cerebellum; DBA, deep brain area*.

* *Statistically significant at p < 0.05*.

**Table 3:** Neuromagnetic peak source strength of interictal and ictal GSWDs.

**Frequency band (Hz)**	**Interictal GSWDs**	**Ictal GSWDs**	***P*-value**
1–4	41.65 ± 26.95	35.91 ± 11.60	0.388
4–8	11.15 ± 4.28	13.37 ± 6.59	0.214
8–12	6.32 ± 5.09	7.13 ± 2.70	0.076
12–30	7.61 ± 5.01	7.89 ± 2.49	0.052
30–80	2.83 ± 2.48	4.35 ± 4.52	0.108
80–250	1.32 ± 2.89	2.54 ± 3.61	0.011
250–500	0.68 ± 1.18	1.45 ± 2.29	0.068

*There was no significant difference after Bonferroni correction for multiple comparisons*.

*GSWD, generalized spike-wave discharge*.

### Functional Network

**Figure 5:**
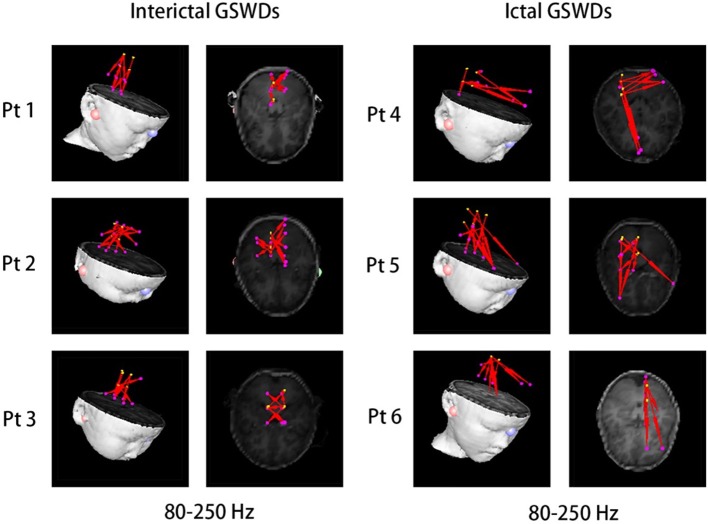
There was significant difference in the functional connectivity (FC) [posterior cingulate cortex (PCC)/precuneus (pC) as the region of interest (ROI)] between interictal and ictal generalized spike-wave discharges (GSWDs) at 80–250 Hz. Interictal GSWDs had FC almost exclusively in the posterior cortex, whereas ictal GSWDs had strong connections in the anterior-posterior pathways (mainly with the frontal cortex).

**Figure 6:**
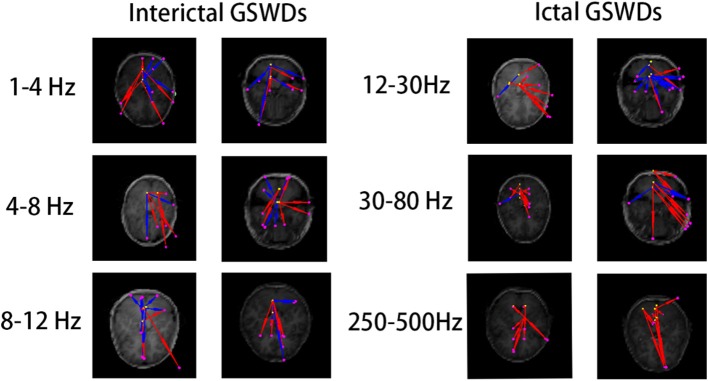
The functional connectivity (FC) [posterior cingulate cortex (PCC)/precuneus (pC) as the region of interest (ROI)] shows no statistical differences between interictal and ictal generalized spike-wave discharges (GSWDs) at 1–4, 4–8, 8–12, 12–30, 30–80, and 250–500 Hz bandwidths.

There were no significant differences in the functional networks of interictal and ictal GSWDs at all frequency bands in the whole brain network when we chose PCC/pC (32) as the ROI. Notably, during interictal GSWD, the majority of CAE patients (12 of 20 segments) showed limited FC in the posterior brain region at 80–250 Hz, whereas the ictal GSWDs (15 of 20 segments) showed mostly strong connections between anterior (particularly in frontal cortex) and posterior regions at the same frequency band. The FC was significantly different between interictal and ictal GSWDs (*p* = 0.001, *p* < 0.05; Figure 5). However, in the other six frequency bands, both the interictal and ictal GSWDs showed FC involving anterior and posterior brain regions. There were no remarkable differences between the two groups in these bandwidths (Figure 6).

## Discussion

We herein studied the frequency-dependent neuromagnetic activities and FC during the interictal and ictal GSWDs within CAE patients using MEG. Our findings have revealed that the magnetic source localization of interictal and ictal GSWDs differs at low and high bandwidths. The FC network involving the PCC/pC showed significant differences at 80–250 Hz between interictal and ictal GSWDs. However, during the preliminary experiment, we found that there was no significant difference between the two groups when the frequency band was over 500 Hz, so the frequency band over 500 Hz was not included in the follow-up study. And there was no statistically significant clinical correlation between the course of epilepsy, drug therapy, and seizures frequency with the corresponding magnetic source localization and FC network in the study.

### Source Localization

Recent EEG-fMRI studies have shown that the frontal cortex, pC, and thalamus are critical for generating absence seizures (31). Our study found significant PCC/pC involvement at 4–8 and 8–12 Hz during interictal GSWDs (Table 2; Figure 2), while there was less PCC/pC involvement during ictal GSWDs at the same bandwidths, thereby suggesting that the interictal neural activities in PCC/pC are higher than ictal at low-frequency bandwidths. A previous EEG study on resting state with low frequency revealed a decreased connectivity with the PCC in temporal lobe epilepsy (40). More recently, a MEG study of CAE patients reported that PCC/pC is markedly decreased in the low-frequency bandwidth in comparison to healthy controls (32). Furthermore, decreased BOLD signaling during GSWDs in the lateral parietal cortices was linear to the duration of EEG discharges (41). These are in agreement with our study. The PCC/pC is involved with rapidly engaging and bilaterally distributed networks for absence seizure (42), as well as the initialization of epileptic activity (36). In a related EEG-fMRI study, IGE showed that the GSWDs and functional change of the PCC may affect one other (27). Moreover, it is also a reportedly critical node in the network that correlates with consciousness in both humans and animals (43). The levels of consciousness may be relative to changes in the activity of the pC. PCC was thought to be playing a key role in the DMN, and the behavioral impairment may relate to the deactivation of pC (44). In addition, the longer the discharge, the more likely the disturbance of consciousness (45). Thus, the neural activity involving the PCC/pC may be associated with the maintenance of consciousness during interictal GSWDs and the disorder of consciousness during ictal GSWDs. Moreover, the lower-frequency pC region is necessary for a sustained and pathological oscillatory state, which is then perpetuated by the corticothalamic network. It is nonetheless possible that the lower-frequency parietal region is representative of inhibition, which has been reported during absence seizures (44).

At the same time, we also found that remarkable MFC source localization at 80–250 Hz during ictal GSWDs (*n* = 25, Table 2; Figure 3), while there was no obvious frontal cortex location during interictal GSWDs at 80–250 Hz. In a recent study of ictal GSWDs during CAE, it is thought that neuromagnetic GSWDs originate from the frontal lobe and thalamus (46). High-frequency oscillation (HFO) has become a new biomarker for epilepsy (47–49) because they are highly localized in the epileptogenic region. We, therefore, speculate that the frontal lobe is the focal point of epilepsy during seizure, and that interictal GSWDs diffuse neural activity generated by the interaction between the cortical and thalamus with no specific focal point. The frontal cortex has previously been demonstrated as a critical element in initiating and propagating absence seizures (34, 36, 46, 50–54) in support of the cortical focus theory (55). These findings altogether suggest that the HFOs of the frontal cortex during ictal GSWDs may be related to the generation of GSWDs (55, 56). In addition, different types of connections and/or information integration at different spatial-temporal levels require different frequencies (57). The frequency of oscillations limits the speed of information transmission. HFOs are better suited to neighboring integration, whereas lower frequencies are used to integrate information from large and/or remote areas (58).

### FC Networks

On the FC networks, there were no obvious differences between the interictal and ictal GSWDs in the whole brain network at different frequency bands. However, when we chose PCC/pC as the seed region, we were surprised to find that, during interictal GSWDs, the networks showed limited FC mostly in the posterior brain region (Figure 5) at 80–250 Hz, whereas the ictal GSWDs showed FC mainly in networks having strong connections between anterior (particularly in frontal cortex) and posterior regions at 80–250 Hz. There were significant differences between the two groups (*p* = 0.001, *p* < 0.05).

The PCC/pC is an important part of the DMN. A study of dynamic causal modeling, for example, reported that pC has a permissive function in the epileptic network for gating GSWDs in absence seizures (27). The SWDs that are generated by the corticothalamic loop are dependent on the state of the pC region (59). It was therefore proposed that the anterior and posterior networks oscillate asynchronously in a normal state and synchronously during transition to an ictal state (44). The posterior cortical regions may also contribute to the earliest activity associated with GSWDs (44). This may explain our finding that FC is mostly limited in the posterior cortical region during interictal GSWD. It has previously been demonstrated that weakened network connections may contribute to preventing hyperexcitability and that attenuation of network connection might be a negative feedback mechanism to prevent CAE seizures (60).

The posterior DMN is likely associated with states of awareness (9, 17, 55, 59, 61). Jing et al. reported that a large number of within- and cross-frequency form dynamics was found in secondary generalization of focal seizures (62). It is therefore possible that a cross-frequency coupling (when slow and fast oscillations interact with one another) produces absence seizures. Seizures with impairment have greater physiological intensity in widespread networks involving much of the brain. Also, comparing to seizures with spared behavior, EEG has shown greater power in widespread brain regions in seizures with impaired behavior (63). These may also explain our findings. However, the connectivity analysis of the virtual sensor activity is based on linear correlation analysis, which is one of the drawbacks of this study.

### Limitations

This study has several limitations. First, the sample size is relatively small owing to the difficulty in collecting interictal data within half an hour of each patient. Second, we did not control for variations in antiepileptic drug (AED) taken by subjects, which could confound MEG recordings. Third, the software used to map the brain is not wholly reliable.

## Conclusions

Our findings have revealed that the magnetic source localization and FC network differ between the interictal and ictal GSWDs in low- and high-frequency ranges. Maintenance of consciousness during interictal GSWDs may be associated with low-frequency activation in the PCC/pC region. Activation of the MFC at frequencies of 80–250 Hz during ictal GSWDs suggests that the frontal cortex is critically involved in propagating CAE. Weakened network connections in interictal GSWDs may be in favor of preventing overexcitability and relates to termination of CAE. The specific mechanisms underlying GSWDs require further study.

## Data Availability Statement

All datasets generated for this study are included in the article/supplementary material.

## Author Contributions

QS analyzed data and wrote this article. TZ, AM, JS, YS, and QC contributed to data analysis. ZH provided the patients. JX provided the MEG software. XW was the general responsible person of the project.

### Conflict of Interest

The authors declare that the research was conducted in the absence of any commercial or financial relationships that could be construed as a potential conflict of interest.

## References

[1] Blumenfeld H. Impaired consciousness in epilepsy. Lancet Neurol. (2012) 11:814–26. 10.1016/S1474-4422(12)70188-6 22898735 PMC3732214

[2] Li Q Luo C Yang T Yao Z He L Liu L. EEG-fMRI study on the interictal and ictal generalized spike-wave discharges in patients with childhood absence epilepsy. Epilepsy Res. (2009) 87:160–8. 10.1016/j.eplepsyres.2009.08.018 19836209

[3] Pittau F Megevand P Sheybani L Abela E Grouiller F Spinelli L Mapping epileptic activity: sources or networks for the clinicians? Front Neurol. (2014) 5:218 10.3389/fneur.2014.00218 25414692 PMC4220689

[4] Seneviratne U Cook M D'Souza W. Focal abnormalities in idiopathic generalized epilepsy: a critical review of the literature. Epilepsia. (2014) 55:1157–69. 10.1111/epi.12688 24938654

[5] Meeren H van Luijtelaar G Lopes da Silva F Coenen A. Evolving concepts on the pathophysiology of absence seizures: the cortical focus theory. Arch Neurol. (2005) 62:371–6. 10.1001/archneur.62.3.371 15767501

[6] Aldenkamp A Arends J. The relative influence of epileptic EEG discharges, short nonconvulsive seizures, and type of epilepsy on cognitive function. Epilepsia. (2004) 45:54–63. 10.1111/j.0013-9580.2004.33403.x 14692908

[7] Arthuis M Valton L Regis J Chauvel P Wendling F Naccache L. Impaired consciousness during temporal lobe seizures is related to increased long-distance cortical-subcortical synchronization. Brain. (2009) 132(Pt 8):2091–101. 10.1093/brain/awp086 19416952

[8] Blumenfeld B Neuroanatomy Through Clinical Cases. Sunderland, MA: Sinauer Associates (2010).

[9] Raichle ME MacLeod AM Snyder AZ Powers WJ Gusnard DA Shulman GL A default mode of brain function. Proc Natl Acad Sci USA. (2001) 98:676–82. 10.1073/pnas.98.2.676 11209064 PMC14647

[10] Broyd SJ Demanuele C Debener S Helps SK James CJ Sonuga-Barke EJ. Default-mode brain dysfunction in mental disorders: a systematic review. Neurosci Biobehav Rev. (2009) 33:279–96. 10.1016/j.neubiorev.2008.09.002 18824195

[11] Gusnard DA Akbudak E Shulman GL Raichle ME. Medial prefrontal cortex and self-referential mental activity: relation to a default mode of brain function. Proc Natl Acad Sci USA. (2001) 98:4259–64. 10.1073/pnas.071043098 11259662 PMC31213

[12] Epilepsia GPJ Generalized epilepsy with spike-and-wave discharge: a reinterpretation of its electrographic and clinical manifestations. The 1977 William G. Lennox Lecture, American Epilepsy Society. Epilepsia. (1979) 20:571–88. 10.1111/j.1528-1157.1979.tb04840.x 477645

[13] Konishi T Matsuzawa J Hongou K Murakami M Yamatani M Yagi S. Partial seizures during the course in patients with absence epilepsy. No To Hattatsu. (1999) 31:395–401. 10487063

[14] Greicius MD Krasnow B Reiss AL Menon V. Functional connectivity in the resting brain: a network analysis of the default mode hypothesis. Proc Natl Acad Sci USA. (2003) 100:253–8. 10.1073/pnas.0135058100 12506194 PMC140943

[15] Fransson P Marrelec G. The precuneus/posterior cingulate cortex plays a pivotal role in the default mode network: evidence from a partial correlation network analysis. Neuroimage. (2008) 42:1178–84. 10.1016/j.neuroimage.2008.05.059 18598773

[16] Aghakhani Y. fMRI activation during spike and wave discharges in idiopathic generalized epilepsy. Brain. (2004) 127:1127–44. 10.1093/brain/awh136 15033899

[17] Gotman J Grova C Bagshaw A Kobayashi E Aghakhani Y Dubeau F. Generalized epileptic discharges show thalamocortical activation and suspension of the default state of the brain. Proc Natl Acad Sci USA. (2005) 102:15236–40. 10.1073/pnas.0504935102 16217042 PMC1257704

[18] Raichle ME Mintun MA. Brain work and brain imaging. Annu Rev Neurosci. (2006) 29:449–76. 10.1146/annurev.neuro.29.051605.112819 16776593

[19] Laufs H Hamandi K Walker MC Scott C Smith S Duncan JS. EEG–fMRI mapping of asymmetrical delta activity in a patient with refractory epilepsy is concordant with the epileptogenic region determined by intracranial EEG. Mag Reson Imaging. (2006) 24:367–71. 10.1016/j.mri.2005.12.026 16677942

[20] Moeller F Siebner HR Wolff S Muhle H Granert O Jansen O. Simultaneous EEG-fMRI in drug-naive children with newly diagnosed absence epilepsy. Epilepsia. (2008) 49:1510–9. 10.1111/j.1528-1167.2008.01626.x 18435752

[21] Carney PW Masterton RA Harvey AS Scheffer IE Berkovic SF Jackson GD. The core network in absence epilepsy. Differences in cortical and thalamic BOLD response. Neurology. (2010) 75:904–11. 10.1212/WNL.0b013e3181f11c06 20702791

[22] Salek-Haddadi A Lemieux L Merschhemke M Diehl B Allen PJ Fish DR. EEG quality during simultaneous functional MRI of interictal epileptiform discharges. Magn Reson Imaging. (2003) 21:1159–66. 10.1016/j.mri.2003.08.017 14725923

[23] Labate A Briellmann RS Abbott DF Waites AB Jackson GD. Typical childhood absence seizures are associated with thalamic activation. Epileptic Disord. (2005) 7:373–7. 16338682

[24] Blumenfeld H. Cellular and network mechanisms of spike-wave seizures. Epilepsia. (2005) 46(Suppl 9):21–33. 10.1111/j.1528-1167.2005.00311.x 16302873

[25] van Luijtelaar G Sitnikova E. Global and focal aspects of absence epilepsy: the contribution of genetic models. Neurosci Biobehav Rev. (2006) 30:983–1003. 10.1016/j.neubiorev.2006.03.002 16725200

[26] Bernhardt BC Rozen DA Worsley KJ Evans AC Bernasconi N Bernasconi A. Thalamo-cortical network pathology in idiopathic generalized epilepsy: insights from MRI-based morphometric correlation analysis. Neuroimage. (2009) 46:373–81. 10.1016/j.neuroimage.2009.01.055 19385011

[27] Vaudano AE Laufs H Kiebel SJ Carmichael DW Hamandi K Guye M. Causal hierarchy within the thalamo-cortical network in spike and wave discharges. PLoS ONE. (2009) 4:e6475. 10.1371/journal.pone.0006475 19649252 PMC2715100

[28] Benuzzi F Mirandola L Pugnaghi M Farinelli V Tassinari CA Capovilla G. Increased cortical BOLD signal anticipates generalized spike and wave discharges in adolescents and adults with idiopathic generalized epilepsies. Epilepsia. (2012) 53:622–30. 10.1111/j.1528-1167.2011.03385.x 22242887

[29] Kotecha R Xiang J Wang Y Huo X Hemasilpin N Fujiwara H. Time, frequency and volumetric differences of high-frequency neuromagnetic oscillation between left and right somatosensory cortices. Int J Psychophysiol. (2009) 72:102–10. 10.1016/j.ijpsycho.2008.10.009 19041674

[30] Gupta D Ossenblok P van Luijtelaar G. Space-time network connectivity and cortical activations preceding spike wave discharges in human absence epilepsy: a MEG study. Med Biol Eng Comput. (2011) 49:555–65. 10.1007/s11517-011-0778-3 21533620

[31] Tenney JR Kadis DS Agler W Rozhkov L Altaye M Xiang J. Ictal connectivity in childhood absence epilepsy: associations with outcome. Epilepsia. (2018) 59:971–81. 10.1111/epi.14067 29633248

[32] Wu C Xiang J Jiang W Huang S Gao Y Tang L. Altered effective connectivity network in childhood absence epilepsy: a multi-frequency MEG study. Brain Topogr. (2017) 30:673–84. 10.1007/s10548-017-0555-1 28286918

[33] Xiang J Wang Y Chen Y Liu Y Kotecha R Huo X. Noninvasive localization of epileptogenic zones with ictal high-frequency neuromagnetic signals. J Neurosurg Pediatr. (2010) 5:113–22. 10.3171/2009.8.PEDS09345 20043746

[34] Tang L Xiang J Huang S Miao A Ge H Liu H. Neuromagnetic high-frequency oscillations correlate with seizure severity in absence epilepsy. Clin Neurophysiol. (2016) 127:1120–9. 10.1016/j.clinph.2015.08.016 26384756

[35] Xiang J Tenney JR Korman AM Leiken K Rose DF Harris E. Quantification of interictal neuromagnetic activity in absence epilepsy with accumulated source imaging. Brain Topogr. (2015) 28:904–14. 10.1007/s10548-014-0411-5 25359158

[36] Miao A Tang L Xiang J Guan Q Ge H Liu H. Dynamic magnetic source imaging of absence seizure initialization and propagation: a magnetoencephalography study. Epilepsy Res. (2014) 108:468–80. 10.1016/j.eplepsyres.2014.01.006 24534760

[37] Xiang J Luo Q Kotecha R Korman A Zhang F Luo H. Accumulated source imaging of brain activity with both low and high-frequency neuromagnetic signals. Front Neuroinf. (2014) 8:57. 10.3389/fninf.2014.00057 24904402 PMC4033602

[38] Xiang J Korman A Samarasinghe KM Wang X Zhang F Qiao H. Volumetric imaging of brain activity with spatial-frequency decoding of neuromagnetic signals. J Neurosci Methods. (2015) 239:114–28. 10.1016/j.jneumeth.2014.10.007 25455340

[39] Miao A Wang Y Xiang J Liu Q Chen Q Qiu W. Ictal source locations and cortico-thalamic connectivity in childhood absence epilepsy: associations with treatment response. Brain Topogr. (2019) 32:178–91. 10.1007/s10548-018-0680-5 30291582

[40] Coito A Genetti M Pittau F Iannotti GR Thomschewski A Holler Y. Altered directed functional connectivity in temporal lobe epilepsy in the absence of interictal spikes: a high density EEG study. Epilepsia. (2016) 57:402–11. 10.1111/epi.13308 26890734

[41] Li Q Zhou D. EEG-fMRI studies on the neural networks of the generalized spike and wave discharges: an overview. Sheng Wu Yi Xue Gong Cheng Xue Za Zhi. (2012) 29:179–83. 22404034

[42] Youssofzadeh V Agler W Tenney JR Kadis DS. Whole-brain MEG connectivity-based analyses reveals critical hubs in childhood absence epilepsy. Epilepsy Res. (2018) 145:102–9. 10.1016/j.eplepsyres.2018.06.001 29936300

[43] Vogt BA Laureys S. Posterior cingulate, precuneal and retrosplenial cortices: cytology and components of the neural network correlates of consciousness. Prog Brain Res. (2005) 150:205–17. 10.1016/S0079-6123(05)50015-3 16186025 PMC2679949

[44] Tenney JR Fujiwara H Horn PS Vannest J Xiang J Glauser TA. Low- and high-frequency oscillations reveal distinct absence seizure networks. Ann Neurol. (2014) 76:558–67. 10.1002/ana.24231 25042348

[45] Sadleir LG Scheffer IE Smith S Carstensen B Carlin J Connolly MB. Factors influencing clinical features of absence seizures. Epilepsia. (2008) 49:2100–7. 10.1111/j.1528-1167.2008.01708.x 18616552

[46] Tenney JR Fujiwara H Horn PS Jacobson SE Glauser TA Rose DF. Focal corticothalamic sources during generalized absence seizures: a MEG study. Epilepsy Res. (2013) 106:113–22. 10.1016/j.eplepsyres.2013.05.006 23764296

[47] Papadelis C Tamilia E Stufflebeam S Grant PE Madsen JR Pearl PL Interictal high frequency oscillations detected with simultaneous magnetoencephalography and electroencephalography as biomarker of pediatric epilepsy. J Vis Exp. (2016) 2016:54883 10.3791/54883 PMC5226354 28060325

[48] Zijlmans M Jiruska P Zelmann R Leijten FS Jefferys JG Gotman J. High-frequency oscillations as a new biomarker in epilepsy. Ann Neurol. (2012) 71:169–78. 10.1002/ana.22548 22367988 PMC3754947

[49] Dumpelmann M Jacobs J Schulze-Bonhage A. Temporal and spatial characteristics of high frequency oscillations as a new biomarker in epilepsy. Epilepsia. (2015) 56:197–206. 10.1111/epi.12844 25556401

[50] Bai X Vestal M Berman R Negishi M Spann M Vega C. Dynamic time course of typical childhood absence seizures: EEG, behavior, and functional magnetic resonance imaging. J Neurosci. (2010) 30:5884–93. 10.1523/JNEUROSCI.5101-09.2010 20427649 PMC2946206

[51] Gupta JR Marsh ED Nieh HA Porter BE Litt B. Discrete gamma oscillations identify the seizure onset zone in some pediatric epilepsy patients. Conf Proc IEEE Eng Med Biol Soc. (2011) 2011:3095–8. 10.1109/IEMBS.2011.6090845 22254994

[52] Szaflarski JP DiFrancesco M Hirschauer T Banks C Privitera MD Gotman J. Cortical and subcortical contributions to absence seizure onset examined with EEG/fMRI. Epilepsy Behav. (2010) 18:404–13. 10.1016/j.yebeh.2010.05.009 20580319 PMC2922486

[53] Tenney JR Fujiwara H Horn PS Rose DF. Comparison of magnetic source estimation to intracranial EEG, resection area, and seizure outcome. Epilepsia. (2014) 55:1854–63. 10.1111/epi.12822 25310937

[54] Westmijse I Ossenblok P Gunning B van Luijtelaar G. Onset and propagation of spike and slow wave discharges in human absence epilepsy: a MEG study. Epilepsia. (2009) 50:2538–48. 10.1111/j.1528-1167.2009.02162.x 19519798

[55] Meeren HK Pijn JP Van Luijtelaar EL Coenen AM Lopes da Silva FH. Cortical focus drives widespread corticothalamic networks during spontaneous absence seizures in rats. J Neurosci. (2002) 22:1480–95. 10.1523/JNEUROSCI.22-04-01480.2002 11850474 PMC6757554

[56] Luttjohann A van Luijtelaar G. The dynamics of cortico-thalamo-cortical interactions at the transition from pre-ictal to ictal LFPs in absence epilepsy. Neurobiol Dis. (2012) 47:49–60. 10.1016/j.nbd.2012.03.023 22465080

[57] Lopes da Silva F Blanes W Kalitzin SN Parra J Suffczynski P Velis DN. Epilepsies as dynamical diseases of brain systems: basic models of the transition between normal and epileptic activity. Epilepsia. (2003) 44(Suppl 12):72–83. 10.1111/j.0013-9580.2003.12005.x 14641563

[58] Engel J Jr da Silva FL. High-frequency oscillations - where we are and where we need to go. Prog Neurobiol. (2012) 98:316–8. 10.1016/j.pneurobio.2012.02.001 22342736 PMC3374035

[59] Masterton RA Carney PW Abbott DF Jackson GD. Absence epilepsy subnetworks revealed by event-related independent components analysis of functional magnetic resonance imaging. Epilepsia. (2013) 54:801–8. 10.1111/epi.12163 23586661

[60] Yang T Luo C Li Q Guo Z Liu L Gong Q. Altered resting-state connectivity during interictal generalized spike-wave discharges in drug-naive childhood absence epilepsy. Hum Brain Mapp. (2013) 34:1761–7. 10.1002/hbm.22025 22431250 PMC6870260

[61] Aru J Aru J Priesemann V Wibral M Lana L Pipa G. Untangling cross-frequency coupling in neuroscience. Curr Opin Neurobiol. (2015) 31:51–61. 10.1016/j.conb.2014.08.002 25212583

[62] Jiang H Cai Z Worrell GA He B. Multiple oscillatory push-pull antagonisms constrain seizure propagation. Ann Neurol. (2019) 86:683–94. 10.1002/ana.25583 31566799 PMC6856814

[63] Guo JN Kim R Chen Y Negishi M Jhun S Weiss S. Impaired consciousness in patients with absence seizures investigated by functional MRI, EEG, and behavioural measures: a cross-sectional study. Lancet Neurol. (2016) 15:1336–45. 10.1016/S1474-4422(16)30295-2 27839650 PMC5504428

